# Survival strategies for the microbiome in a vent-dwelling glass sponge from the middle Okinawa Trough

**DOI:** 10.3389/fmicb.2025.1636046

**Published:** 2025-08-29

**Authors:** Yu-Hang Li, Ming Yang, Tao-Shu Wei, Hua-Guan Chen, Lin Gong, Yong Wang, Zhao-Ming Gao

**Affiliations:** ^1^State Key Laboratory of Deep-Sea Science and Intelligent Technology, Institute of Deep-sea Science and Engineering, Chinese Academy of Sciences, Sanya, China; ^2^Department of Deep-Sea Science, Institute of Deep-Sea Science and Engineering, Chinese Academy of Sciences, Sanya, China; ^3^University of Chinese Academy of Sciences, Beijing, China; ^4^Hainan Deep-Sea Technology Innovation Center, Sanya, China; ^5^Institute of Oceanology, Chinese Academy of Sciences, Qingdao, China; ^6^Institute for Ocean Engineering, Shenzhen International Graduate School, Tsinghua University, Shenzhen, China; ^7^HKUST-CAS Sanya Joint Laboratory of Marine Science Research, Chinese Academy of Sciences, Sanya, China

**Keywords:** deep sea, hydrothermal vent, symbiosis, methanotrophy, glass sponge

## Abstract

The adaptive mechanisms of sponge microbiomes to harsh deep-sea environments, including hydrothermal vents and cold seeps, remain unclear. Here, we used metagenomics to investigate the microbiome of an undescribed vent-dwelling glass sponge from the middle Okinawa Trough, probably representing a novel species within the family Bolosominae. Eleven high-quality prokaryotic metagenome-assembled genomes (MAGs) were retrieved, none assignable to known species, with two representing new genera. Dominant MAGs included sulfur-oxidizing bacteria (SOB) and ammonia-oxidizing archaea, followed by methane-oxidizing bacteria (MOB) and nitrite-oxidizing bacteria. Global distribution analysis suggested that most MAGs were sponge-specific symbionts. Comparative genomics revealed functional redundancy among SOB and early-stage genome reduction in a unique MOB lineage. Additionally, a total of 410 viral contigs were identified, most exhibiting a lytic lifestyle and forming distinct clades from known viruses. Our work expands understanding of the diversity and novelty of deep-sea sponge-associated prokaryotes and viromes, and suggests their niche adaptation to hydrothermal fluid environments.

## 1 Introduction

Marine sponges in the phylum Porifera are ancient metazoans that emerged on the earth at 600 ~ 890 million years ago (Turner, 2021; Yin et al., 2015). They are widely distributed through the global ocean (Van Soest et al., 2012) and commonly host dense and diverse communities of microorganisms (Thomas et al., 2016; Turner, 2021). These sponge-associated microbiomes, comprising prokaryotes, protists, fungi, microalgae and viruses, can constitute up to 40% of the sponge biomass (Webster and Taylor, 2012). Symbiotic microorganisms are thought to play crucial roles in sponge evolution and ecological success, forming a stable and functional unit together with the host, known as a holobiont (Webster and Thomas, 2016). Compared to their free-living relatives, sponge symbionts exhibit specific adaptations to symbiotic lifestyles, such as genome streamlining, loss of motility, and an enrichment of genes encoding eukaryotic-like proteins and involved in defense systems (Gao et al., 2014; Horn et al., 2016; Reynolds and Thomas, 2016). These symbionts also contribute significantly to nutrient cycling within the holobiont system (Burgsdorf et al., 2022; Moeller et al., 2023; Zhang et al., 2019). Because of their ancient origin and ecological importance, sponge holobionts have drawn great attention (Anupama et al., 2023).

Sponge holobionts in shallow marine environments have been extensively studied (Pita et al., 2018). With the advancement of deep-sea sampling technologies, sponges have also been successfully collected from the deep ocean, and their associated microbiomes have been investigated using both 16S rRNA amplicon sequencing and metagenomic approaches (Busch et al., 2022; Wei et al., 2023). For instance, analyses of 13 phylogenetically diverse deep-sea sponge species belonging to the Demospongiae and Hexactinellida from the South Pacific revealed that, although their bacterial communities were broadly similar to those of shallow-water sponges, their archaeal communities were predominantly composed of ammonia-oxidizing genera within the family Nitrosopumilaceae (Steinert et al., 2020). A more comprehensive study revealed that two-thirds of all known bacterial phyla have been discovered in deep-sea sponges, with the most common members affiliated with the phyla Pseudomonadota, Chloroflexota, Acidobacteriota, and Bacteroidota (Busch et al., 2022). Metagenomic studies further illustrated that chemoautotrophs including sulfur-oxidizing bacteria (SOB), ammonia-oxidizing archaea (AOA), and nitrite-oxidizing bacteria (NOB), are the main players in deep-sea sponges and could benefit the sponge hosts by providing complementary nutrients (Tian et al., 2016; Wei et al., 2023; Zhou et al., 2019). Genomic profiles of Thaumarchaeota AOA revealed close associations with both depth and symbiosis (Garritano et al., 2023; Steinert et al., 2020; Wang et al., 2022). Remarkably, a recent study reported that methane-oxidizing bacteria (MOB) can act as symbiotic partners of deep-sea sponges from asphalt seeps, and these MOB exhibit genome reduction compared to their free-living relatives (Rubin-Blum et al., 2019). Viruses are the most abundant entities in the ocean and have also been implicated in regulating prokaryotic community dynamics and promoting nutrient cycling (Roux et al., 2016; Suttle, 2007). Recent studies suggest that bacteriophages played important roles within sponge holobionts, thereby attracting growing scientific attention (Nguyen et al., 2021; Pascelli et al., 2018; Zhou et al., 2021). Despite these advances, our knowledge about sponges and their associated microbiomes remains limited in the deep sea, especially in extreme environments such as hydrothermal vents and cold seeps.

The Okinawa Trough is a geologically complex deep-sea region that harbors at least 25 hydrothermal vents and multiple cold seeps (Mars Brisbin et al., 2020; Miyazaki et al., 2017). A wide range of benthic fauna in this region relies on chemoautotrophic symbionts to adapt to the heterogeneous environmental conditions shaped by fluid diffusion, depth, and vent and seep distributions (Mars Brisbin et al., 2020). Zhou et al. (2021) characterized the sponge-associated microbiomes and virus-symbiont interactions in three deep-sea demosponges from hydrothermal vent fields in the southern Okinawa Trough. In this study, we report a previously undescribed deep-sea glass sponge species collected from a hydrothermal vent site in the middle Okinawa Trough, and present a comprehensive analysis of its associated microbiome. We successfully retrieved 11 prokaryotic metagenome-assembled genomes (MAGs), evaluating their taxonomic novelty, global distribution and metabolic potential. Furthermore, viral sequences were identified, and the ecological functions of these potential phages were explored. This work expands our understanding of the complexity and ecological relevance of virus-microbe-sponge interactions in deep-sea chemosynthetic ecosystems.

## 2 Methods

### 2.1 Sample collection

A sponge individual (hereafter named as SPSG) was collected from a hydrothermal vent site (depth 1,284 m, 126°58.49′ E 27°32.46′ N) at the Iheya North field in the middle Okinawa Trough using ROV Discovery on board the R/V Kexue in April 2014. Upon arrival at the main deck of the R/V, specimens were rinsed with 0.22 μm membrane-filtered iced seawater to remove contaminated microbes. The cleaned specimens were cut into small pieces with a sterile razor blade, preserved in 100% ethanol and stored at −80°C for metagenomic analyses.

### 2.2 Metagenomic DNA extraction and sequencing

A 0.5 cm^3^ volume of sponge tissue was transformed into 1 mL of DNA extraction buffer (50 mM Tris–HCl, 40 mM EDTA, 500 mM NaCl, 0.75 M sucrose, pH = 8) and fully cut into tiny pieces with sterile scissors. The suspension was subjected to DNA extraction with the PowerSoil DNA Isolation Kit (MoBio Laboratories, Carlsbad, CA, USA) following the default experimental procedure. The extracted DNA was quantified using the Qubit dsDNA HS Assay Kit with Qubit 2.0 Fluorometer (Invitrogen, Carlsbad, CA, USA) and stored at −80°C for further processing. Qualified genomic DNA samples, with an input amount of 100 ng, were randomly interrupted using the Covaris M200 Autofocus Sonic Genome Shear instrument (Covaris, Massachusetts, USA). A metagenomic library was constructed using the TruSeq^®^ Nano DNA LT Kit (Illumina, San Diego, CA, USA) and sequenced on the HiSeq2500 platform to generate 2 × 150 bp paired-end reads. An additional metagenomic library for in-depth sequencing was constructed using the same DNA sample through the VAHTS^®^ Universal DNA Library Prep Kit for Illumina V4 (Illumina, San Diego, CA, USA) and sequenced on the NovaSeq X Plus platform to generate 2 × 150 bp paired-end reads.

### 2.3 Metagenome assembly and genome recovery

Two sequencing runs were performed, yielding a total of 24.9 Gb raw data. Quality control of raw reads was conducted using fastp v0.23.2 (Chen, 2023) with the parameters “-D -g -l 50 -q 20 -u 40 -W 3 -c -3” to trim adapters, filter low-quality reads, and remove duplicates. These two quality-controlled datasets were separately assembled and co-assembled using both MEGAHIT v1.2.9 (Li et al., 2015) with a kmer list of “21, 29, 39, 59, 79, 99, 119, 141” and SPAdes v3.13.0 (Nurk et al., 2017) with default settings for metagenomic assembly. Genome binning was performed on the assembled metagenomes using MetaWRAP v1.2 (Uritskiy et al., 2018) with default parameters to generate MAGs. Redundant MAGs were dereplicated using dRep v3.4.2 (Olm et al., 2017). The quality of the resulting MAGs was evaluated using CheckM v1.2.2 (Parks et al., 2015) and their taxonomic classification was conducted using GTDB-Tk v2.3.2 (Chaumeil et al., 2019) against the GTDB database R214 (Parks et al., 2022).

### 2.4 Microbial relative abundance and global distribution

The relative abundance of a symbiont within the sponge microbiome was estimated as the percentage of reads mapped to its corresponding MAG relative to the total metagenomic dataset, after filtering out eukaryotic reads. At first, metagenomic contigs longer than 1000 bp were subjected to EukRep v.0.6.7 (West et al., 2018) for identifying eukaryotic contigs. Subsequently, metagenomic reads were mapped to the eukaryotic contigs by Bowtie2 (Langmead and Salzberg, 2012), and the mapped reads were removed from the metagenomic dataset. Finally, the remaining reads were subjected to CoverM v0.2.0^1^ to calculate the relative abundance of symbiotic MAGs. The global distribution of sponge symbionts was analyzed using datasets in the Sponge Microbiome Project (SMP) (Moitinho-Silva et al., 2017) and the Deep Sea Sponge Microbiome Project (D-SMP) (Busch et al., 2022). The 16S rRNA gene sequences of sponge symbionts were searched against these two datasets using BLAST v2.14.0 + (Camacho et al., 2009) with an e-value threshold of “1e-05.” Information on the target sequences was extracted to calculate their relative abundance in respective sponges and adjacent seawater samples.

### 2.5 Genome annotation

Close relatives of the sponge symbionts were annotated through the program GTDB-Tk and used as reference genomes. Open reading frames of the symbiotic MAGs and their relatives were predicted using Prokka v1.14.5 (Seemann, 2014). The predicted genes were annotated using KofamScan v1.3.0 (Aramaki et al., 2020) against the KEGG database 109.0 (Kanehisa et al., 2016), eggNOG-emapper v2.1.4 (Cantalapiedra et al., 2021) against the eggNOG database 5.0 (Huerta-Cepas et al., 2019), and PfamScan v1.0 (Madeira et al., 2022) against the Pfam database v36.0 (Mistry et al., 2021). These MAGs were also annotated using antiSMASH v7.0 (Blin et al., 2023) for potential secondary metabolic gene clusters. Reference genomes with more than 90% completeness were selected for comparative genomic analyses, and orthologous genes (OGs) shared by MAGs were summarized using OrthoFinder v2.5.5 (Emms and Kelly, 2019). All orthologous genes were annotated subsequently as mentioned above. The average nucleotide identity (ANI) values and the average amino identity (AAI) values between sponge symbionts and their relatives were calculated using pyani v0.2.12 (Pritchard et al., 2016) and CompareM v0.1.2,^2^ respectively.

### 2.6 Phylogenetic inference

For preliminary sponge identification, the *coxI* gene encoding cytochrome c oxidase subunit I was retrieved from metagenomic contigs and its closely related sequences were downloaded from the NCBI GenBank database. Multiple sequence alignment of *coxI* genes was generated using MAFFT (Katoh and Standley, 2013), and was trimmed using trimAl v1.4.1 (Capella-Gutiérrez et al., 2009). A maximum likelihood (ML) phylogenetic tree was constructed using IQ-TREE v2.1.4 (Minh et al., 2020) with parameter settings: “-MFP -bootstrap 1000.” Species from the class Demospongiae were used as the outgroup. For phylogenetic analyses of sponge symbionts, 16S rRNA genes were predicted using Meta_RNA (Huang et al., 2009) and their closely related genes were downloaded from the NCBI GenBank database and the SILVA database 138.1 (Pruesse et al., 2007). A ML tree was constructed using the same methods mentioned above. For phylogenetic analysis, the alignment of 43 concatenated conserved protein-coding genes deduced from symbiotic MAGs and their relatives was retrieved using the CheckM program, trimmed with trimAl (Capella-Gutiérrez et al., 2009), and subsequently used to construct a ML tree with IQ-TREE (Minh et al., 2020).

### 2.7 Virus identification and annotation

Viral-like contigs (VLCs) were firstly identified using VirSorter2 v2.2.4 (Guo et al., 2021), and false positive fragments were removed through several filtering steps (Zhou et al., 2022). Firstly, VLCs were annotated based on a built-in database of VirSorter2 for viral genes. A positive VLC should have at least two viral specific genes (e.g., genes containing terminase, phage tail, head, etc.), or contained viral sequences and had more than 70% of genes annotated with hypothetical/unknown function. Secondly, VLCs with annotated prokaryotic specific genes (e.g., ribosomal genes) will also be removed. Thirdly, the basic characteristics of VLCs were assessed using CheckV v1.0.1 (Nayfach et al., 2021). CheckV could identify proviruses (lysogenic virus) and remove the host sequence regions in proviruses. Subsequently, the VLCs were clustered into viral operation taxonomic units (vOTUs) using CD-HIT v4.8.1 (Li and Godzik, 2006) with the parameters: “-c 0.95-n 10 -aS 0.85 -d 0.” Potential viral-host linkages were predicted using iPHoP v1.3.3 (Roux et al., 2023). In addition, a comprehensive strategy was employed to identify virus-host linkages, integrating multiple principles such as homology-based matching, CRISPR spacer sequence alignment, and k-mer frequency similarity between viral and host genomes (Pascelli et al., 2020). The relative abundance of the vOTUs was calculated using CoverM with the “-contig” mode (Langmead and Salzberg, 2012). A gene-sharing network between vOTUs and reference sequences (ProkaryoticViralRefSeq207-Merged) was generated using vConTACT2 v.0.11.34 (Bin Jang et al., 2019). These vOTUs were also loaded into VirSorter2 to generate the required input files for DRAM-v v1.3.5 (Shaffer et al., 2020), which was subsequently executed to annotate potential auxiliary metabolic genes (AMGs) with default settings.

### 2.8 Statistics and reproducibility

Statistical analyses and data visualization were primarily constructed using packages in R v4.4.3. Bar charts, box plots, heat maps, and stacked bar plots were generated with the package *ggplot2* v3.4.4. Venn diagrams were constructed using the package *VennDiagram* v3.3.1 via the “draw.quintuple.venn” function. In addition, viral gene-sharing networks and the virus-host interactions were visualized using Cytoscape v3.10.0 (Shannon et al., 2003).

## 3 Results

### 3.1 Sponge identification

**Figure 1:**
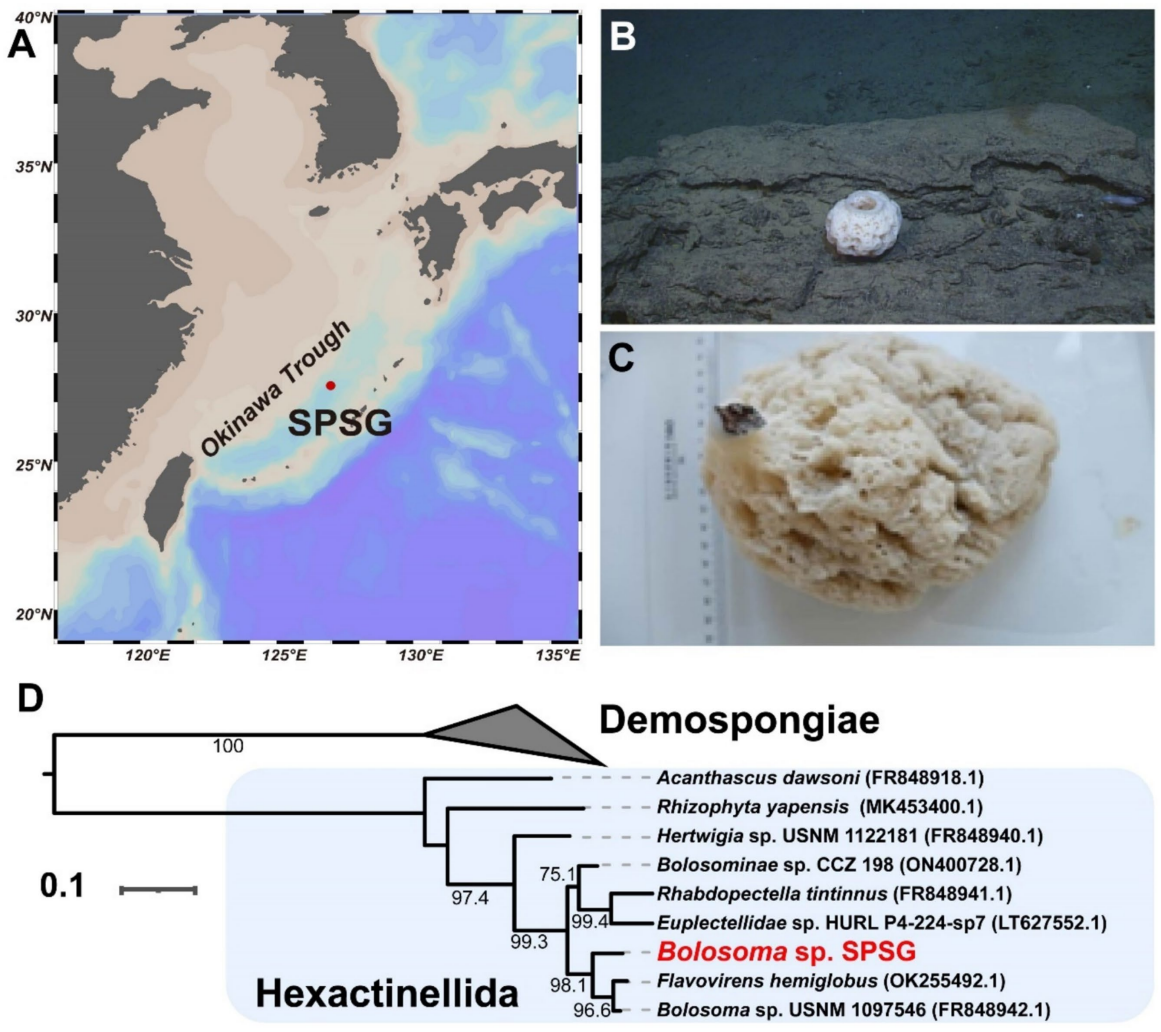
A novel vent-dwelling sponge from the middle Okinawa Trough. **(A)** Sampling location of the sponge individual (SPSG) was marked as a red dot. **(B)** *In situ* photograph of SPSG. **(C)** Sponge specimen in the lab. **(D)** The coxI-based Maximum-Likelihood phylogenetic tree. The tree is constructed using IQ-TREE2 with the “TIM2 + F + I + G4” model and bootstrap value was set to 1,000. The scale bar represents 0.1 substitutions per nucleotide position.

A white, brain-like sponge was collected from carbonate sedimentary rocks in a hydrothermal vent field in the middle Okinawa Trough (Figures 1A–C). Phylogenetic inference based on the *cox1* gene (1,728 bp) suggested that our sponge was closely related to *Bolosoma* sp. USNM 1097546 and *Flavovirens hemiglobus*, both of which belong to the class Hexactinellida (Figure 1D). The sponge shared 93.3% sequence identity with the *cox1* gene of the closest related species *Bolosoma* sp. USNM 1097546. In COI meta-barcoding studies, a variety of sequence similarity cutoffs were used ranging from 95 to 100% to result in species-like groupings (Porter and Hajibabaei, 2020). Our sample thus was considered to represent a novel sponge species. Preliminary morphological analyses revealed that our species attached directly by its basal part, although its microscleres had only discoidal outer ends, which was very different from known *Bolosoma* species that always have a peduncle. As the species could not be resolved at the genus level, it was designated at the subfamily level and named *Bolosominae* sp. SPSG.

### 3.2 Prokaryotic genome recovery

Eleven prokaryotic MAGs with estimated completeness greater than 95% and contamination less than 5% were successfully retrieved from the sponge metagenome (Table 1). The estimated genome sizes of these MAGs range from 1.44 to 5.09 Mb, with GC contents varying between 31.33 and 58.10%. Taxonomic classification using GTDB-Tk assigned these MAGs to four phyla: Thermoproteota (*n* = 2), Nitrospinota (*n* = 1), Omnitrophota (*n* = 1), and Pseudomonadota (*n* = 7). All the MAGs potentially represent new species, as none could be assigned to known species-level taxa. Notably, Omnitrophota Bin05 and Pseudomonadota Bin12 are proposed to represent new genera, based on their low relative evolutionary divergence (RED) values of 0.84 and 0.78, respectively (Parks et al., 2018) (Supplementary Table S1).

**Table 1:** General genomic features of sponge-associated prokaryotic MAGs.

**MAG**	**Phylum** ^**a**^	**Genome size (bp)**	**GC (%)**	***N***_**50**_ **(bp)**	**No. of contigs**	**Longest contig**	**Comp. (%)** ^**b**^	**Contam. (%)** ^**b**^	**RED** ^**c**^
Bin01	Thermoproteota	1,553,672	32.35	196,904	15	385,213	99.55	0.01	0.99
Bin02	Thermoproteota	1,610,942	31.33	55,621	77	166,890	98.19	0	0.99
Bin04	Nitrospinota	3,011,330	49.85	850,697	5	888,025	95.61	0.61	0.97
Bin05	Omnitrophota	1,757,838	40.74	9,053	272	28,024	95.55	1.77	0.84
Bin06	Pseudomonadota	3,769,389	58.10	980,110	6	1,868,960	96.51	0.79	0.96
Bin08	Pseudomonadota	5,091,673	43.06	129,049	70	526,459	97.65	0.48	0.92
Bin09	Pseudomonadota	3,396,869	37.26	33,821	221	98,190	97.38	0.34	0.90
Bin10	Pseudomonadota	1,441,324	40.21	46,592	48	125,553	100	0.01	0.90
Bin11	Pseudomonadota	1,579,913	40.41	153,804	13	383,677	100	0	0.93
Bin12	Pseudomonadota	4,929,865	55.28	1,336,792	16	1,545,215	97.9	0.28	0.78
Bin13	Pseudomonadota	2,180,910	41.47	2,180,910	1	2,180,910	98.8	0.03	0.97

^a^The novel revision of the phylum nomenclature was based on the List of Prokaryotic names with Standing in Nomenclature (LPSN) (https://lpsn.dsmz.de/). ^b^Genome completeness (Compl.) and contamination (Contam.) were estimated by CheckM (Parks et al., 2015). ^c^RED, relative evolutionary divergence that indicates taxonomic novelty.

**Figure 2:**
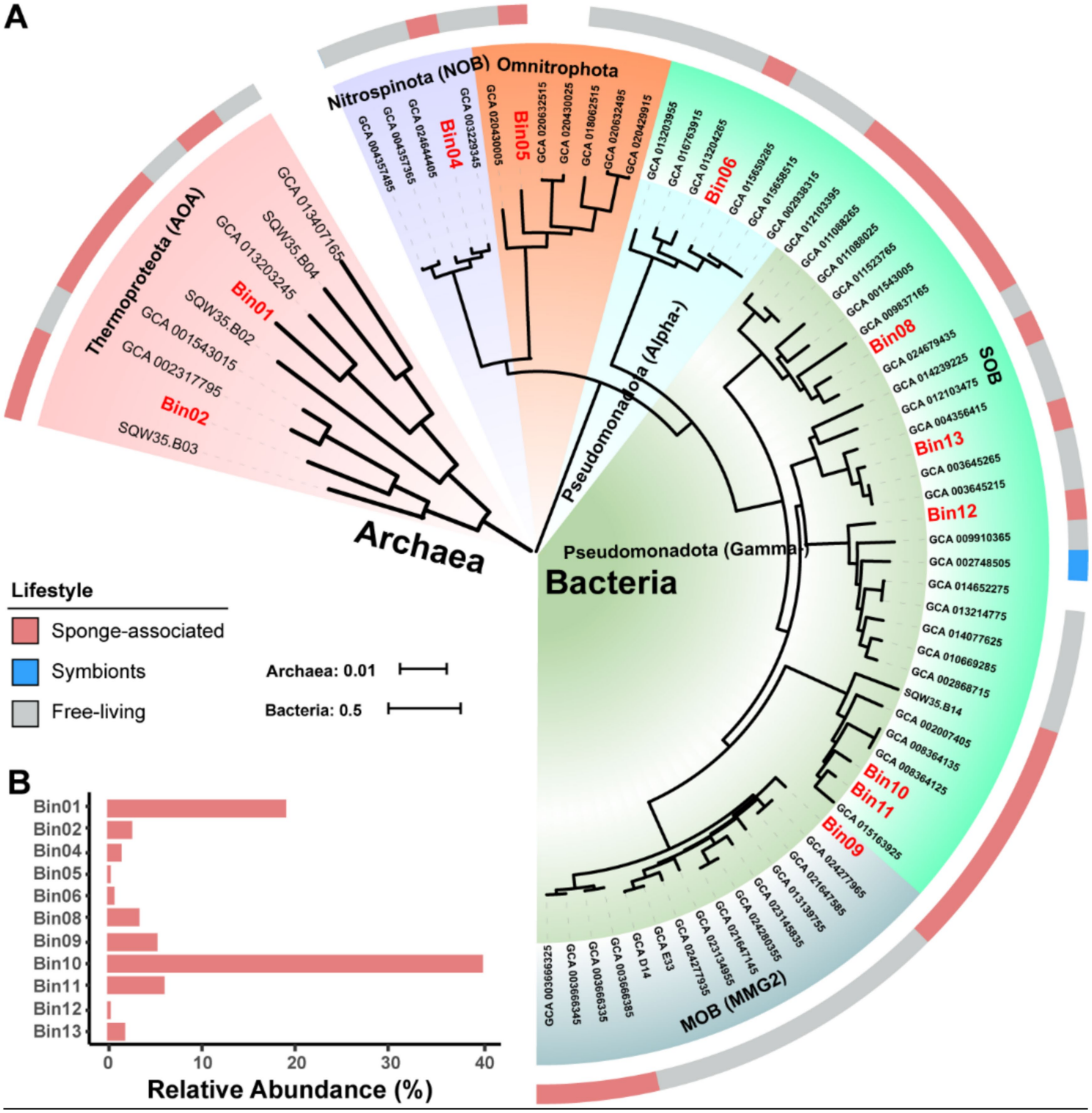
Phylogenomic tree of sponge-associated MAGs and their relative abundance. **(A)** Maximum-Likelihood phylogenomic tree of the archaeal MAGs is constructed based on a concatenated alignment of 53 conserved archaeal proteins using the “Q.plant + F + I + R2” model. Maximum-Likelihood phylogenomic tree of the bacterial kingdom is constructed based on a concatenated alignment of 120 conserved bacterial proteins using the “LG + F + R5” model. Both archaeal and bacterial conserved protein sets are deduced from the GTDB-Tk program. Sponge-associated MAGs in this study are labeled with red color. **(B)** Relative abundance of MAGs in the sponge microbiome. The relative abundance is calculated with coverM v0.2.0 using metagenome reads after eliminating reads assigned to eukaryotic contigs. MAG, metagenome-assembled genome; AOA, ammonia-oxidizing archaea; NOB, nitrite-oxidizing bacteria; SOB, sulfur-oxidizing bacteria; MOB, methane oxidizer bacteria; Alpha-, Alphaproteobacteria; Gamma-, Gammaproteobacteria.

Phylogenomic inference revealed that all the MAGs formed distinct and separated lineages (Figure 2A and Supplementary Table S2), and shared ANI values below 92.02% and AAI values below 92.29% with known species (Supplementary Tables S3, S4), further supporting their taxonomic novelty (Konstantinidis et al., 2017). MAGs Bin01 and Bin02 fell into the genus *Nitrosopumilus* of the phylum Thermoproteota, a common AOA group widely detected in seawaters, sediments and sponges (Garritano et al., 2023; Wei et al., 2023), with Bin01 sharing an ANI of 91.62% with a free-living strain GCA_013203245 and Bin02 sharing an ANI of 92.02% with a sponge-associated strain GCA_001543015. Bin04 was affiliated with NOB in the phylum Nitrospinota, typically coupling with AOA to complete nitrification in sponges (Tian et al., 2016). It shared the highest ANI value (88.18%) with a free-living relative bacterium GCA_003229345. Bin05 belonged to parasitic nanobacteria within the phylum Omnitrophota (Seymour et al., 2023), a group not previously reported in sponges. It shared notably low ANI and AAI values with its closest relatives, suggesting high divergence. Bin06 was clustered together with SOB in the genus Casp-alpha2 of the class Alphaproteobacteria, sharing a maximum ANI of 88.58% with a free-living relative Rhodospirillales bacterium GCA_015659285. Bin08 was affiliated with the genus LS-SOB in the phylum Pseudomonadota and shared the highest ANI of 76.81% with a sponge-associated relative *Candidatus Spongiihabitans thiooxidans* GCA_001543005. Bin09 shared the highest ANI (77.47%) with a methane-oxidizing sponge symbiont GCA_003666385, and classified within the family Methylomonadaceae of the phylum Pseudomonadota (Rubin-Blum et al., 2019). Bin10 and Bin11 were affiliated with the genus JAAOIF01 (corresponding to the *SUP05* clade in the Silva database), a well-known SOB group in hydrothermal ecosystem and also widely associated with sponges (Zhou et al., 2019). Bin12 was affiliated with the family Halieaceae in the phylum Pseudomonadota, sharing an ANI of 76.35% and an AAI of 63.22% with its closest relative *Parahalioglobus pacificus* GCA_014652275. Bin13 was closely related to the genus SMWN01 in the phylum Pseudomonadota and shared an ANI of 81.71% with Gammaproteobacteria bacterium GCA_003645215.

To reveal the components of the sponge microbiome, we calculated the relative abundance of these MAGs using normalized sequencing depth (Figure 2B and Supplementary Table S1). The total reads mapped to 11 MAGs accounted for 81.35% of all the metagenomic reads, indicating that our retrieved MAGs represent the sponge microbiome very well. The resulting relative abundances ranged from 0.21% (Omnitrophota Bin05) to 39.81% (Pseudomonadota SOB Bin10). Among the classified taxa, the Pseudomonadota (57.98%) and Thermoproteota (21.47%) were the two most dominant prokaryotic phyla, with the Pseudomonadota Bin10 (39.81%) and the Thermoproteota AOA Bin01 (18.95%) being the most abundant MAGs, respectively. Functionally, based on the relative abundance of MAGs, sulfur-, ammonia-, and methane-oxidizing symbionts represented 52.62, 21.47, and 5.35% of the microbial community, respectively.

### 3.3 Global distribution patterns

**Figure 3:**
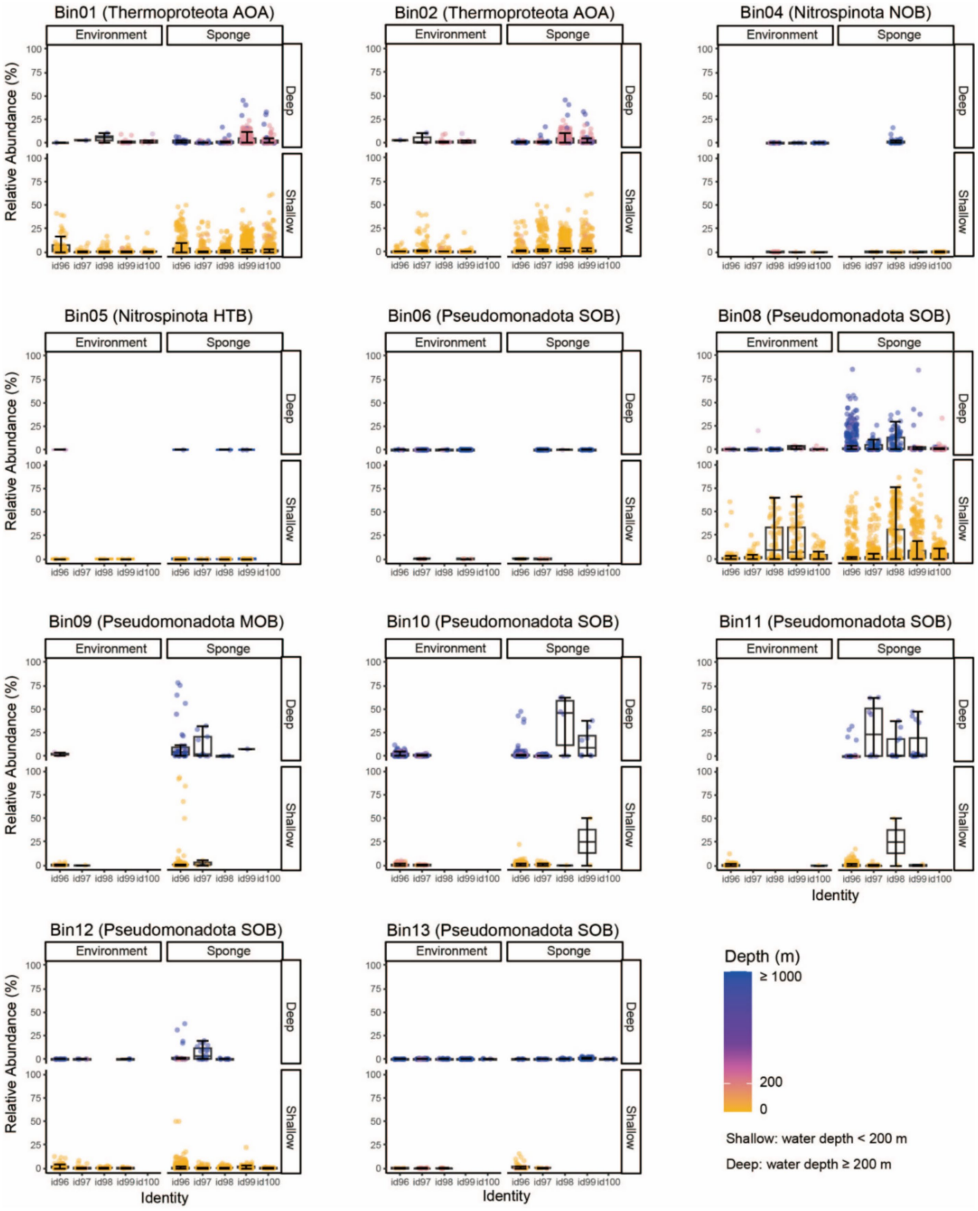
Global distribution patterns of sponge-associated MAGs. Predicted 16S rRNA gene sequences of sponge-associated MAGs are searched against 16S rRNA amplicons in the SMP and D-SMP datasets. Global distribution patterns are drawn through summarizing the relative abundance of targeted sequences in respective samples. “Environment/Sponge” indicates the sample source. “id” Indicates identity, e.g., id96 means the 16S rRNA gene shared an identity of more than 96% and less than 97% with its relatives. Colors indicate the sampling depths of reference sequences. MAG, metagenome-assembled genome; SMP, Sponge Microbiome Project; D-SMP, Deep Sea Sponge Microbiome Project; AOA, ammonia-oxidizing archaea; NOB, nitrite-oxidizing bacteria; HTB, heterotrophic bacteria; SOB, sulfur-oxidizing bacteria; MOB, methane oxidizer bacteria.

To determine the global distribution patterns of sponge-associated microbes, putative 16S rRNA gene sequences of all 11 MAGs were successfully retrieved from both binned genomes and metagenome-assembled contigs (Supplementary Table S5), and were subsequently queried against the SMP and D-SMP databases (Figure 3 and Supplementary Table S6). According to the distribution patterns, sponge microbes could be grouped into generalists (being distributed in a wide range of sponge species) and specialists (living in a small number of sponge species) (Haber et al., 2021). Our analyses indicated that seven MAGs comprising two Thermoproteota AOAs (Bin01 and Bin02), one Nitrospinota NOB (Bin04) and four Pseudomonadota SOBs (Bin06, Bin08, Bin12, Bin13) fell into the generalist group, of which their relatives (≥ 99% identity) could be detected in large number of sponge and environmental samples. The remaining four MAGs (Omnitrophota Bin05, and Pseudomonadota Bin09, Bin10 and Bin11) were probably specialists, which are found in a limited number of sponges and rarely detectable in environmental samples.

In the generalist group, the closest relatives of the highly abundant Thermoproteota AOA Bin01 and Bin02 (≥99% identity) occupied high proportions in both deep and shallow sponges, whereas the relative abundance of their closest environmental relatives was relatively low. The closest relatives of Pseudomonadota SOB Bin08 (≥99% identity) showed low relative abundance in deep sponge and environmental samples. However, more distant relatives (96 ~ 98% identity) were highly abundant in deep sponge samples. Notably, the relatives of Bin08 could reach high abundance in both shallow sponge and environmental samples. Given that the amplicons in the SMP database target only the V3 region of the 16S rRNA gene and were limited to 100 bp in length, the resulting low taxonomic resolution hindered accurate discrimination between the query sequences and their closest relatives. As a result, the above three highly abundant generalists could only be tentatively considered sponge-specific taxa or species that had adapted to the sponge habitat, pending further high-resolution confirmation. The closest relatives of Pseudomonadota SOB Bin12 were found in shallow sponges, yet their distribution patterns between sponge and environmental samples are unclear. Since the far distant relatives showed higher abundance in sponge samples than in environmental samples, Bin12 might still represent a sponge-adaptive species. Alternatively, the close relatives of Nitrospinota NOB Bin04, Pseudomonadota SOB Bin06 and Bin13 were consistently low abundance and their distribution showed no obvious differences between sponge and environmental samples. Thus, all these three species probably belonged to opportunistic microbes, but might have some connection with the sponge hosts.

In the specialist group, the closest relatives of putative Pseudomonadota MOB Bin09 and Pseudomonadota SOB Bin10 and Bin11 were endemic to sponge samples, and the relatives of Bin09 and Bin10 (≥98% identity) were only detectable in deep sponges. Thus, the three MAGs should belong to sponge-specific species. The close relatives of heterotrophic Omnitrophota Bin05 (≥99% identity) in 19 sponge and two environmental samples were consistently low abundance, thus probably represented an opportunistic microbe.

### 3.4 Sponge-associated microbial central metabolisms and eukaryotic-like proteins

**Figure 4:**
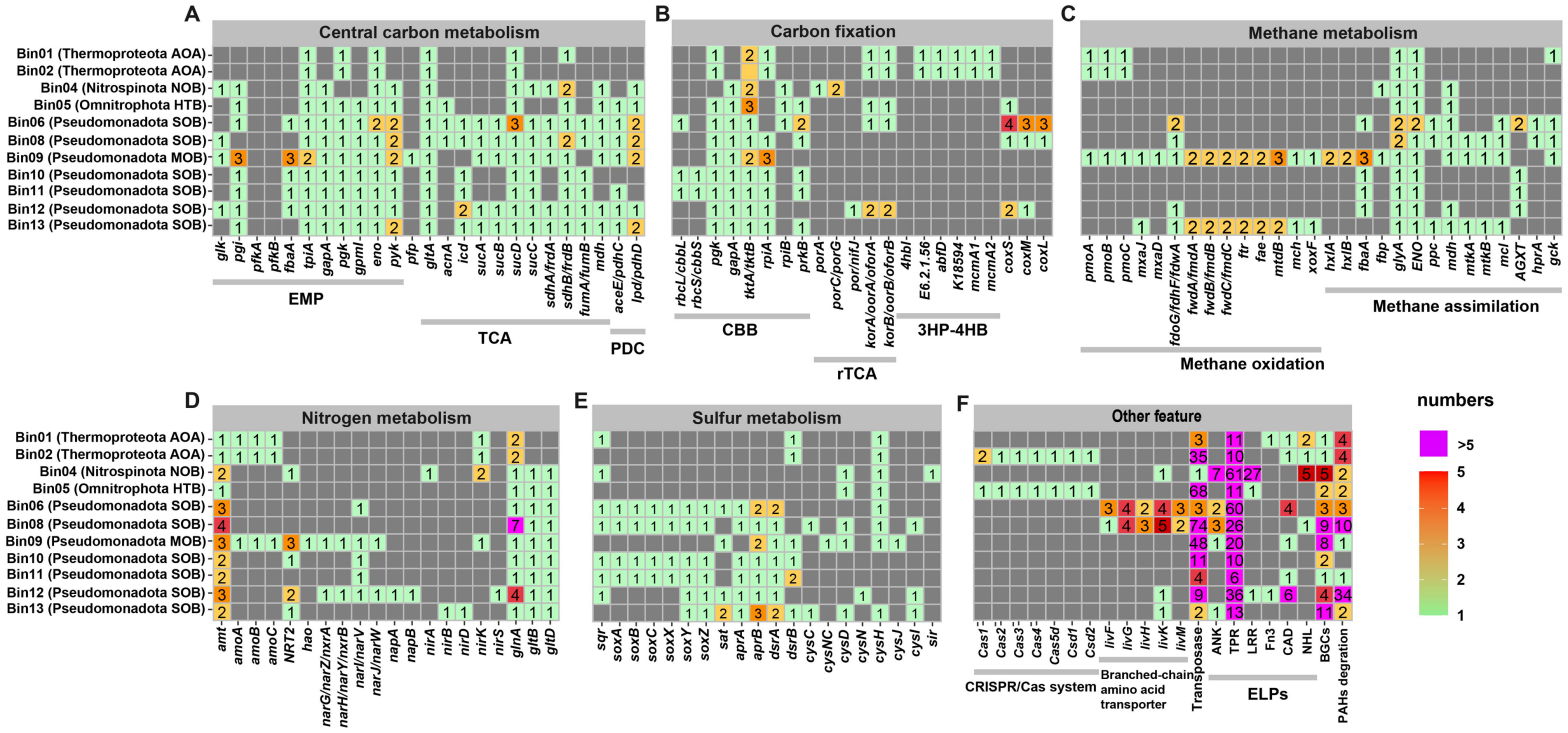
Metabolic potential and adaptive features of sponge-associated microbes. The colored blocks indicate the presence of related genes, and gray blocks indicate the absence of genes. Copy numbers of genes were marked with different colors. AOA, ammonia-oxidizing archaea; NOB, nitrite-oxidizing bacteria; HTB, heterotrophic bacteria; MOB, methane oxidizer bacteria; SOB, sulfur-oxidizing bacteria; CBB, Calvin-Benson-Bassham cycle; rTCA, reverse tricarboxylic acid cycle; 3HP-4HB, 3-hydroxypropionate/4-hydroxybutyrate cycle; EMP, Embden-Meyerhof-Parnas Pathway; TCA, tricarboxylic acid cycle; PDC, Pyruvate Dehydrogenase Complex; ELPs, eukaryotic like proteins.

To comprehensively explore the potential ecological functions of sponge-associated microbial communities, we annotated and summarized key pathways related to carbon, nitrogen, and sulfur metabolism, as well as functional genes involved in CRISPR/Cas systems, branched-chain amino acid transport and eukaryotic-like proteins (ELPs) (Figures 4A–E and Supplementary Table S7).

Among these pathways, core carbon metabolism showed notable variation across the microbial genomes. All these sponge-associated microbial genomes contained an incomplete glycolysis pathway, primarily due to the absence of *pfk* genes encoding 6-phosphatefructokinase (Figure 4A). The Pseudomonadota SOB MAGs Bin06, Bin08, Bin12 and Bin13 possessed essential genes related to the tricarboxylic acid (TCA) cycle, while the other MAGs, except for Pseudomonadota MOB Bin09, lacked the key gene *sucA*, which encodes 2-oxoglutarate dehydrogenase. Thermoproteota AOA Bin01 and B02 were capable of carbon fixation via the 3-hydroxypropionate/4-hydroxybutyrate (3HP-4HB) cycle, as evidenced by the presence of key genes encoding 3-hydroxypropionyl-CoA synthetase (K18594) and 4-hydroxybutyrate-CoA ligase (E6.2.1.56) (Figure 4B). The Pseudomonadota SOB Bin06, Bin10 and Bin11 possessed key genes associated with the Calvin-Benson-Bassham (CBB) cycle, including *cbbL*, *pgk*, and *prkB*, suggesting their potential for autotrophic carbon fixation via this pathway. In contrast, none of the analyzed genomes, including the putative Nitrospinota NOB Bin04, harbored a complete set of genes for the reductive citric acid (rTCA) cycle, suggesting the absence of this alternative carbon fixation mechanism. Pseudomonadota MOB Bin09 contained a *pmoABC* operon encoding the methane monooxygenase and a *xoxF* gene encoding lanthanide-dependent methanol dehydrogenase (Figure 4C), suggesting its potential to oxidize methane to formaldehyde. Additionally, Bin09 harbored genes related to formaldehyde assimilation via the ribulose monophosphate (RuMP) pathway (*hxlA*, *hxlB*, and *fba*A), as well as genes involved in formaldehyde oxidization to carbon dioxide for energy production (*fae*, *mtd*, *mch*, *ftr*, *fwd*, and *fdh*). Interestingly, Pseudomonadota SOB Bin06 and Bin08 possessed the *coxSLM* operon for carbon monoxide oxidization, while Pseudomonadota SOB Bin12 contained two of these subunits.

Genes related to nitrogen metabolism were mainly found in Thermoproteota AOAs (Bin01 and Bin02), and in Pseudomonadota MOB (Bin09) and SOBs (Bin12 and Bin13) (Figure 4D). Both Bin01 and Bin02 possessed the *amoABC* genes encoding the subunits of ammonia monooxygenase, however, the *hao* gene, which encodes hydroxylamine oxidoreductase, were not detected. Bin09 and Bin12 harbored genes involved in denitrification, including those encoding the membrane-bound respiratory nitrate reductase (*narGHIJ*), the periplasmic dissimilatory nitrate reductase (*napAB*), and NO-forming nitrite reductase (*nirK*/*nirS*), indicating their potential to converts nitrate to nitric oxide. Bin13 contained the *nirBD* genes encoding nitrite reductase, which are associated with the dissimilatory nitrate reduction pathway. All bacterial MAGs possessed genes related to ammonia transportation (*amt*) and assimilation (*glnA*, *gltBD*). Similarly, the archaeal MAGs Bin01 and Bin02 contained *amt* and *glnA* genes. Moreover, five bacterial MAGs, including one Nitrospinota NOB (Bin04), one Pseudomonadota MOB (Bin09), and three Pseudomonadota SOB (Bin10, Bin12, and Bin13), harbored the NRT2 gene encoding nitrate/nitrite transporter.

The vent-dwelling sponge-associated microbes in the phylum Pseudomonadota were strongly associated with sulfur oxidization (Figure 4E). Four Pseudomonadota SOB (Bin06, Bin08, B10 and Bin11) had the *sat* gene encoding sulfate adenylyltransferase, the *aprAB* genes encoding adenylylsulfate reductase and the *dsrAB* genes encoding dissimilatory sulfite reductase, which comprised of the dissimilatory sulfate reduction pathway for reversely oxidizing sulfide to sulfate. Three Pseudomonadota SOB (Bin06, Bin12 and Bin13) contained complete *soxABCXYZ* gene sets encoding the sulfur oxidation complex (SOX) for thiosulfate oxidation. Another three Pseudomonadota SOB (Bin08, B10 and Bin11) harbored the *soxABXYZ* gene sets, yet did not contain the *soxC* gene. Besides, seven MAGs including Bin10 and B11 had the *sqr* gene encoding sulfide-quinone oxidoreductase that could transform sulfide to sulfur globules. Genes related to assimilatory sulfate reduction (such as *cysH* and *cysD*) were also present in several MAGs.

In addition, several eukaryotic-like domain proteins (ELPs) were identified in these sponge-associated MAGs (Figure 4F). All MAGs encoded tetratricopeptide repeats (TPRs), with Nitrospinota NOB Bin04 and Pseudomonadota SOB Bin06 each containing over 100 TPRs. Five of the nine bacterial MAGs, including one Nitrospinota NOB Bin04, one Pseudomonadota NOB Bin09, and three Pseudomonadota SOB (Bin06, Bin08 and Bin13), harbored genes encoding ankyrin repeats (ANKs), whereas neither of the two Thermoproteota AOA (Bin01 and Bin02) contained ANKs. Bin04 had up to 27 genes encoding leucine rich repeats (LRRs), while heterotrophic Omnitrophota Bin05 and Pseudomonadota SOB Bin12 were the only other MAGs with a single LRR each. The two Thermoproteota AOA possessed extra genes encoding cadherin domain (CADs), Fibronectin type III domain (Fn3), and NHL repeats (NHLs). Among bacterial genomes, CADs were found in four Pseudomonadota (Bin06, Bin09, Bin11 and Bin12), whereas Fn3 and NHLs were rarely detected.

### 3.5 Comparative analyses of six putative SOB symbionts

In total, two AOA and six SOB were identified (Supplementary Table S1). Comparative genomic analysis revealed limited functional differences between two AOA (Supplementary Table S8), whereas these SOB exhibited substantial genomic divergence. Among the six sulfur-oxidizing Pseudomonadota, a total of 3,153 OGs were identified, but only 666 (21.12%) were shared among all six genomes, representing the core OGs (Supplementary Figure S1 and Supplementary Table S9). Accessory genes accounted for 56.11% (1,769) of total OGs. The species-specific genes of these Pseudomonadota SOB (Bin06, Bin08, Bin10/11, Bin12 and Bin13) were 117, 186, 187, 206 and 22, respectively. In particular, key genes in the CBB cycle, such as *cbbL* encoding the large subunit of ribulose-1,5-bisphosphate carboxylase/oxygenase, *gapA* encoding glyceraldehyde-3-phosphate dehydrogenase and *prkB* encoding phosphoribulokinase, were observed in Bin06, Bin10 and Bin11, implying their autotrophic capabilities. In contrast, the complete *livFGHKM* associated with branched-chain amino acid transport and indicative of heterotrophic potential (Haber et al., 2021), was identified in the MAGs Bin06 and Bin08. Furthermore, Bin12 harbored an array of genes related to the degradation of toluene, phenol, steroid, and other aromatic compounds, suggesting potential roles in detoxifying polycyclic aromatic hydrocarbons (PAHs) and supporting sponge survival in contaminated environments (Marzuki et al., 2021). Although Bin08 also encoded ten genes related to PAH degradation, the corresponding pathways were considerably less complete compared to Bin12 (Figure 4 and Supplementary Table S7).

### 3.6 Phylogenetic and functional inference of the putative MOB symbiont

**Figure 5:**
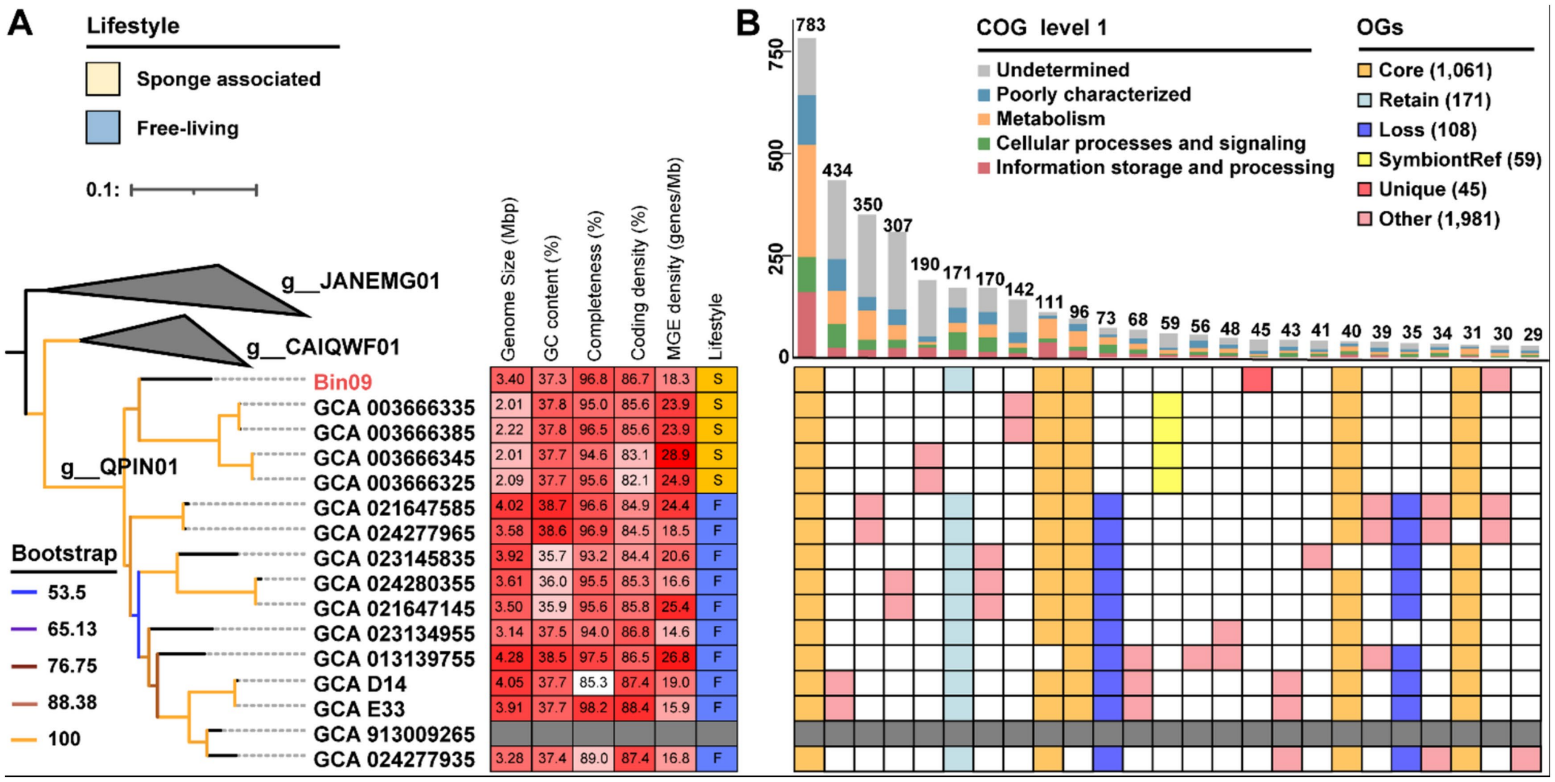
Phylogenomic and functional inferences of the putative MOB symbiont. **(A)** Maximum-likelihood phylogenomic tree of Pseudomonadota Bin09 was constructed based on a concatenated alignment of 43 CheckM-derived conserved proteins using the “Q.plant + I + R3” model. Genome size, GC content, completeness, coding density and transposable element density of Bin09 and its relatives are given. In the annotation of lifestyle, “S” and “F” represent symbiotic and free-living lifestyles, respectively. **(B)** Upset diagram shows shared and unique genes among Bin09 and its symbiotic and free-living relatives. Core, core OGs of all the analyzed genomes. “Retain,” OGs shared by free-living relatives and Bin09. “Loss,” OGs only detected in free-living relatives. “SymbiontRef,” OGs only shared by symbiotic relatives. “Uniq,” the unique OGs of Bin09. MOB, methane-oxidizing bacteria; MGE, mobile genetic element; OG, orthologous gene.

Phylogenomic analysis placed the putative Pseudomonadota MOB Bin09 within the genus-level clade QPIN01 of the family Methylomonadaceae, forming a distinct and distant branch adjacent to four sponge-associated symbionts from the asphalt seeps of the Gulf of Mexico (Rubin-Blum et al., 2019) (Figure 5A). This suggested that Bin09 likely represented an individual lineage, which was consistent with its undetermined species-level classification by the GTDB-Tk program. The genome of Bin09 was 3.40 Mb in size, larger than those of its symbiotic relatives (2.01 ~ 2.22 Mb), but smaller than most free-living counterparts (3.50 ~ 4.28 Mb) except GCA_023134955 (3.14 Mb) and GCA_024277935 (3.28 Mb). Its coding density was 86.7%, moderately higher than that of all relative symbiotic genomes (82.1% ~ 85.7%), yet not obviously different from that free-living reference genomes (84.4% ~ 88.4%). Its GC content of 37.26% did not differ significantly from either symbiotic (37.7% ~ 37.8%) or free-living relatives (35.7% ~ 38.7%). Alternatively, Bin09 harbored 62 mobile genetic elements (MGEs)-related genes, corresponding to a MGE density of 18.3 elements per Mb, which is lower than that of many free-living MOBs and more comparable to values observed in symbiotic MOB genomes (Supplementary Table S1).

Comparative genomic analyses were conducted on Pseudomonadota MOB Bin09 and its high-quality relatives (completeness > 90%) within the same genus (Figure 5B). Bin09 shared 1,061 core OGs with all analyzed genomes. This total included genes absent in only one of the free-living relatives, comprising 783, 111, 96, 40 and 31 OGs, respectively. Moreover, Bin09 shared 171 accessory genes exclusively with free-living relatives (termed “Retain” OGs), while it lacked 108 accessory genes that were present only in their symbiotic relatives (termed “Loss” OGs). There were 45 unique OGs belonging to Bin09 (termed “Unique” OGs). COG-based functional analyses illustrated that the “Retain” genes were primarily involved in signal transduction mechanisms [category T], cell motility [N] and unknown function [S] (Supplementary Table S10 and Supplementary Figure S2). The “Loss” genes were mainly related to inorganic ion transport and metabolism [P], transcription [K], cell mobility [N], and energy production and conversion [C]. Notably, the retained [N] category included genes related to all functional components of the flagellar and pilus systems, whereas the lost [N] category comprised genes encoding components of the flagellar export apparatus of the type III secretion system. The lost [P] category also harbored genes involved in the phosphate transport system. Most of the “Unique” genes could not be functionally annotated.

### 3.7 Virus identification and functional analyses

**Figure 6:**
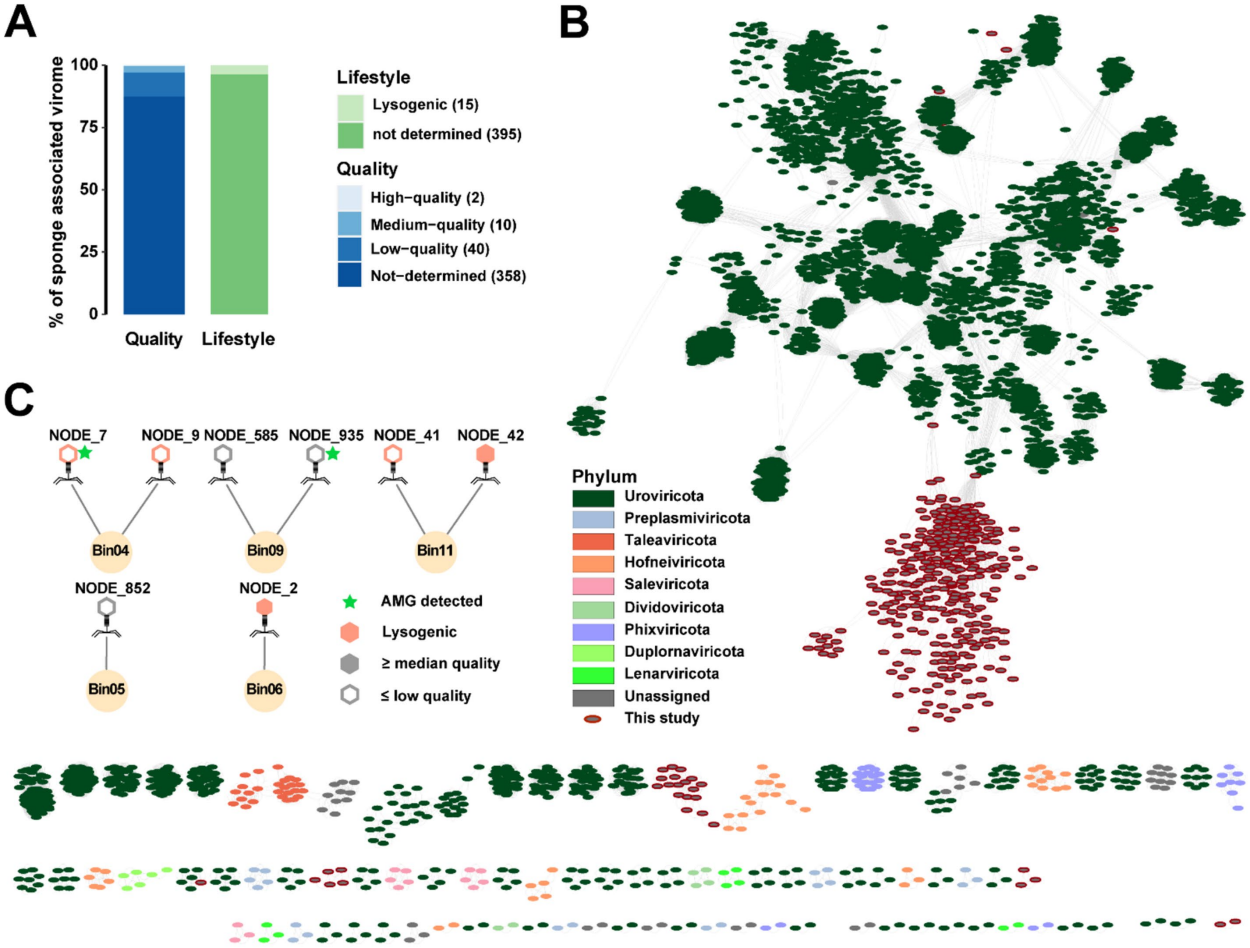
Viral gene-sharing networks and virus-host linkages. **(A)** Bar charts showing the lifestyle and quality of predicted viral contigs. **(B)** Gene-sharing networks produced with vConTACT2. Each circle indicates a viral contig (vOTU), and their taxonomies are colored. **(C)** Viruses and their potential prokaryotic hosts. Lysogenic viruses are marked with orange color. Virus-encoding AMGs are marked with light green stars.

SPAdes-assembled contigs were screened for viruses, yielding 417 viral candidates clustered into 410 vOTUs (Figure 6A and Supplementary Table S11). Based on the CheckV evaluation, the vOTUs included two high-quality, ten medium-quality, 358 low-quality and 40 not-determined entries. The two high-quality vOTUs represented the longest viral sequences, with lengths of 51,399 bp and 48,949 bp, respectively (Supplementary Table S11). CheckV analysis further indicated that three medium-quality and twelve low-quality vOTUs likely undergo a lysogenic lifestyle. Gene-sharing networks illustrated that the sponge-associated viruses were highly distinct from known viral taxa (Figure 6B). iPHoP analyses identified eight phages that were probably to interact with five prokaryotic hosts based on homology match (Figure 6C and Supplementary Table S12). These host-associated phages included two medium-quality, five low-quality and one not-determined phages, of which five are likely prophages. The two medium-quality phages predicted to be associated with Pseudomonadota Bin06 and Bin11. Totally, 48 AMGs were identified from 38 low-quality/not-determined vOTUs (Supplementary Table S13 and Supplementary Figure S3). The most prevalent AMGs included those encoding phosphate starvation-inducible protein (PF02562, PhoH) and 2OG-Fe(II) oxygenase superfamily (PF13640), which were found in twelve and five vOTUs, respectively. Additionally, two AMGs of the sulfotransferase family (PF13469) and one encoding DsrC-like protein (PF04358) were associated with sulfur metabolism. One AMG encoding cobaltochelatase (CobS, K09882) might be involved in cobalamin biosynthesis.

## 4 Discussion

### 4.1 Novelty of the vent-dwelling sponge microbiome

Chemosymbiosis is a key adaptive strategy employed by invertebrates such as siboglinid tubeworms, vesicomyid clams, and bathymodiolin mussels to thrive in harsh deep-sea environments, including hydrothermal vents and cold seeps (Sogin et al., 2021). Conversely, members of the phylum Porifera, despite their global distribution and ecological significance, are rarely observed in these extreme environments. To date, only a few seep-dwelling and vent-sponge species have been reported (Wang et al., 2023; Zhou et al., 2021). Zhou et al. (2021) had analyzed the microbiome of several sponge species from the southern Okinawa Trough, yet they mainly focused on the virus-host interactions. Thus our knowledge of the diversity and novelty of sponge microbiomes in vent environments remains limited. Here, we report the discovery of a previously undescribed vent-dwelling glass sponge species, which likely belongs to a new genus. Metagenomic analysis yielded 11 prokaryotic MAGs, none of which could be assigned to known species. Notably, two of these MAGs probably represented novel genus-level sponge symbionts. Biogeographical patterns further support the uniqueness of these sponge-specific symbionts. Additionally, we characterized the sponge-associated virome, identifying 410 vOTUs, the majority of which lacked affiliation with known viral taxa. In summary, our findings expand the known diversity of glass sponges inhabiting harsh deep-sea environments and underscore the remarkable novelty and complexity of their associated microbiomes. This work highlights the underexplored diversity of sponge-associated microbial communities and underscores the need for further studies on sponges from extreme deep-sea ecosystems.

### 4.2 Metabolic plasticity and redundancy of the sponge symbionts

Deep-sea sponge microbiomes are typically dominated by AOA and SOB, with NOB playing complementary roles in transforming nitrite produced by AOA to nitrate (Steinert et al., 2020; Tian et al., 2016). Fluids from hydrothermal regions in Iheya North are enriched with reduced compounds such as hydrogen sulfide, methane and hydrogen (Miyazaki et al., 2017; Chen et al., 2025). These geochemical properties provide a chemically reduced environment that supports diverse chemosynthetic microbial communities. The microbiome of the vent-dwelling sponge described in this study includes not only dominant AOA and SOB symbionts but also MOB, which is inconsistent with previous studies of prokaryotic communities in a large number of deep-sea sponge species (Busch et al., 2022; Wei et al., 2023). This finding likely reflects adaptation to the specific geochemical conditions of the Iheya North hydrothermal field. The presence of multiple chemoautotrophs may enable the host to efficiently utilize available energy sources, supporting the development of dense sponge populations observed in ecosystems such as those in the southern Okinawa Trough and the Formosa Ridge cold seep (Wang et al., 2023; Zhou et al., 2021). Interestingly, this vent sponge hosts six Pseudomonadota SOB symbionts (Bin06, Bin08, and Bin10-13) that fall into five distinct clades. Four of them (Bin06, Bin08, Bin10 and Bin11) contain complete dissimilatory sulfate reduction pathways that allow for the reverse oxidation of sulfide to sulfate. Among them, Bin06, Bin10 and Bin11 also carry essential genes for carbon fixation via the CBB cycle. Notably, Bin06 and Bin08 possess the complete *livFGHKM* operon, which encodes branched-chain amino acid transporters. Both also likely utilize carbon monoxide through the *coxSLM* operon (Gao et al., 2019). In contrast, Bin12 and Bin13 contain intact *soxABCXYZ* gene sets, encoding the SOX complex for thiosulfate oxidation. Additionally, Bin12 carries a rich set of genes associated with PAH degradation. These findings suggest that different SOB clades exhibit functional specification and may occupy different ecological niches within the sponge holobiont. Future investigations incorporating metatranscriptomic and proteomic data are needed to clarify their ecological roles within the sponge holobiont. Overall, this study reveals metabolic plasticity and redundancy of vent-dwelling sponge microbiomes, which likely supports their survival in dynamic and disturbed deep-sea environments (Pita et al., 2018).

### 4.3 A putative MOB sponge symbiont under early genome reduction

MOB are well known for their symbiotic relationships with bathymodiolin mussels in hydrothermal vents and cold seeps. Recent studies have expanded the recognized range of MOB hosts, such as the vent snail *Gigantopelta aegis* and deep-sea feather duster worms (Goffredi et al., 2020; Lan et al., 2021). Additionally, two deep-sea sponges from asphalt seeps have been found to derive nutrition from symbiotic MOB (Rubin-Blum et al., 2019). Building on these findings, our work presents the second genome-level study of sponge-MOB partnerships, and notably, the first one originating from a hydrothermal vent environment. Phylogenomic inference and GTDB-based taxonomic classification reveal that the methane-oxidizing Pseudomonadota symbiont Bin09 represents a distinct lineage, unaffiliated with any currently known species, thereby highlighting the previously unrecognized diversity of sponge-associated MOB. Bin09 possesses a relatively larger genome size than seep-derived MOB symbionts but is smaller than most of free-living relatives. Functional analyses further showed that Bin09 had lost several gene sets compared to its free-living counterparts, particularly those related to cell motility. Nevertheless, it retains a substantial number of orthologous genes shared with free-living strains (the “Retain” OGs), suggesting that Bin09 is undergoing an early stage of genome reduction (Gao et al., 2023). The high proportion of transposase-encoding genes provides additional support for this hypothesis (McCutcheon and Moran, 2012). These findings raise intriguing questions about the evolutionary trajectory of MOB symbionts in deep-sea sponges. Notably, 16S rRNA analysis has demonstrated that six sponge species from the Formosa Ridge cold seep displayed a significant genetic divergence both from each other and from previously characterized taxa (Wang et al., 2023). Future genome-resolved metagenomic studies focusing on sponge-MOB partnerships may yield deeper insights into their diversity, evolution and ecological roles.

### 4.4 Conceptual framework of nutrient cycles within sponge holobiont

Viruses are the most abundant entities in the ocean, playing key roles in shaping microbial communities and mediating biogeochemical cycles through nutrient release and gene transfer (Roux et al., 2016; Suttle, 2007). Phage-host dynamics are commonly explained by two ecological models: the “Kill-the-Winner” and “Piggyback-the-Winner” hypotheses (Chen et al., 2021). In the former, lytic phages infect and lyse dominant microbes, reducing their abundance and recycling nutrients, whereas the latter suggests that temperate phages favor lysogeny under high host density, integrating into host genomes without immediate lysis. Recent studies have revealed high diversity and host-specificity of viral communities in sponge holobionts (Jahn et al., 2021; Pascelli et al., 2020). In the vent-dwelling sponge from the Okinawa Trough, five putative lysogenic phages predicted to be associated with low abundance MAGs. In contrast, the two predominant MAGs, Thermoproteota AOA Bin01 and Pseudomonadota SOB Bin10, did not host detectable lysogenic phages. While the quality of the viral dataset and the reliance on a single host prediction method limit the robustness of this observation, this pattern may tentatively reflect a “Kill-the-Winner” dynamic, in which dominant hosts are more frequently targeted by lytic phages (Chen et al., 2021). Further investigation using higher-quality viral metagenomic data is required to validate this hypothesis.

**Figure 7:**
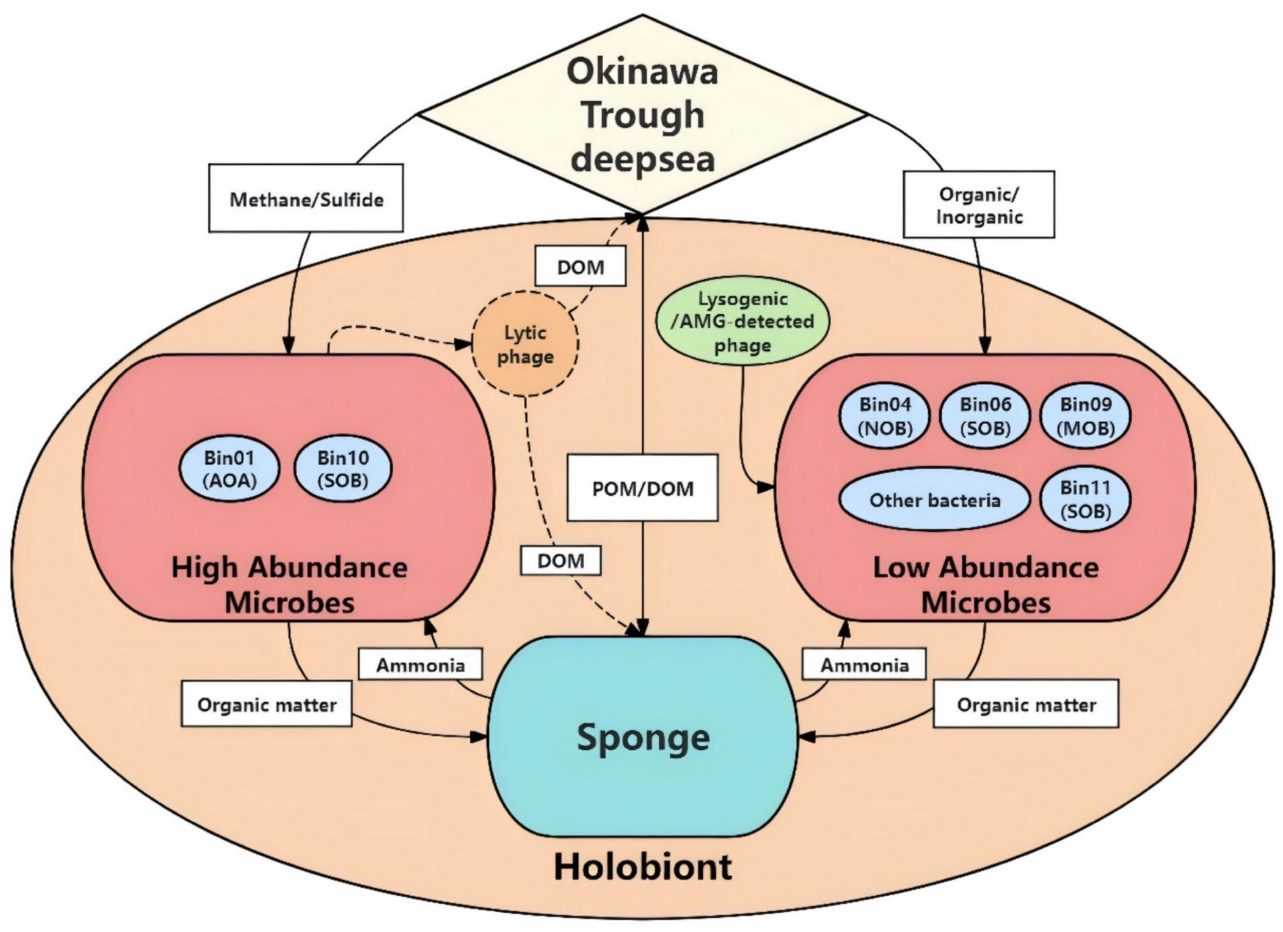
Conceptual framework of nutrient cycles in the sponge holobiont. AOA, ammonia-oxidizing archaea; SOB, sulfur-oxidizing bacteria; NOB, nitrite-oxidizing bacteria; MOB, methane oxidizer bacteria; POM, particulate organic matter; DOM, dissolved organic matter.

Finally, we propose a conceptual framework illustrating the interactions among sponge, their symbiont, and associated viruses within the vent-dwelling sponge holobiont (Figure 7). The sponge pumps hydrothermal vent fluids in the middle Okinawa Trough, acquiring methane and hydrogen sulfide, which serve as energy sources for its symbiotic microorganisms. Chemoautotrophic symbionts, including SOB and MOB, fix inorganic carbon and generate energy to support both their own metabolism and that of the sponge host. The host sponge assimilates nutrients derived from both symbionts and pumped seawaters, and in turn, produces metabolic waste, including ammonia. This waste is mitigated by AOA and NOB, which help maintain nitrogen balance within the holobiont. Additionally, low-abundance members such as Pseudomonadota SOB Bin12 contribute to detoxification by degrading extra aromatic hydrocarbons. Microbial communities could be modulated by viruses, which appear to follow a lytic lifecycle by infecting predominant symbionts, thereby regulating microbial community structure and promoting nutrient cycling through the “Kill-the-Winner” pattern. In contrast, viruses linked to low-abundance symbionts are lysogenic and promote host survival and fitness through AMGs. This integrated system demonstrates a complex but coordinated interplay that facilitates holobiont stability and resilience in a chemically dynamic vent ecosystem.

## 5 Conclusion

The Okinawa Trough, where a large number of seeps and vents have been reported (Wu et al., 2022), is an ideal model for studying deep-sea chemosynthetic ecosystems. Here, we report an undescribed glass sponge species in the family Bolosominae, and extensively characterize its microbiome, including prokaryotes and viruses. We reveal sponge-specific distribution patterns and functional redundancy and novelty of prokaryotic symbionts. We highlight five SOB as redundant nutrient producers and a methanotrophic symbiont undergoing early genome reduction. We also demonstrate the indispensable roles of phages on the sponge holobiont. Our work extends the knowledge about the adaptive mechanism of sponge microbiomes to deep-sea extreme ecosystems, and improves the understanding of the establishment and maintenance process of sponge holobionts. However, due to sampling limitations, geochemical data and information on sponge population density were not obtained. The sample was preserved in ethanol, which prevented metatranscriptomic analysis. Future research cruises may provide a more comprehensive understanding of this vent-dwelling sponge species.

## Data Availability

The raw metagenomic data and prokaryotic MAGs from the vent-dwelling sponge are available via the NCBI database under the BioProject PRJNA1261396.

## Footnotes

^1^ https://github.com/wwood/CoverM

^2^ https://github.com/dparks1134/CompareM
