# Supplementary material for: Survival strategies for the microbiome in a vent-dwelling glass sponge from the middle Okinawa Trough
Source: Front Microbiol. 2025 Aug 29;16:1636046. doi: 10.3389/fmicb.2025.1636046 (PMC12426252; doi:10.3389/fmicb.2025.1636046)

Supplementary Material

# Supplementary Figures

**Supplementary Figure S1.** OGs shared within sponge-associated SOB symbionts. OGs were produced by OrthoFinder. Abbreviations: OGs, orthologous genes; SOB, sulfur-oxidizing bacteria.


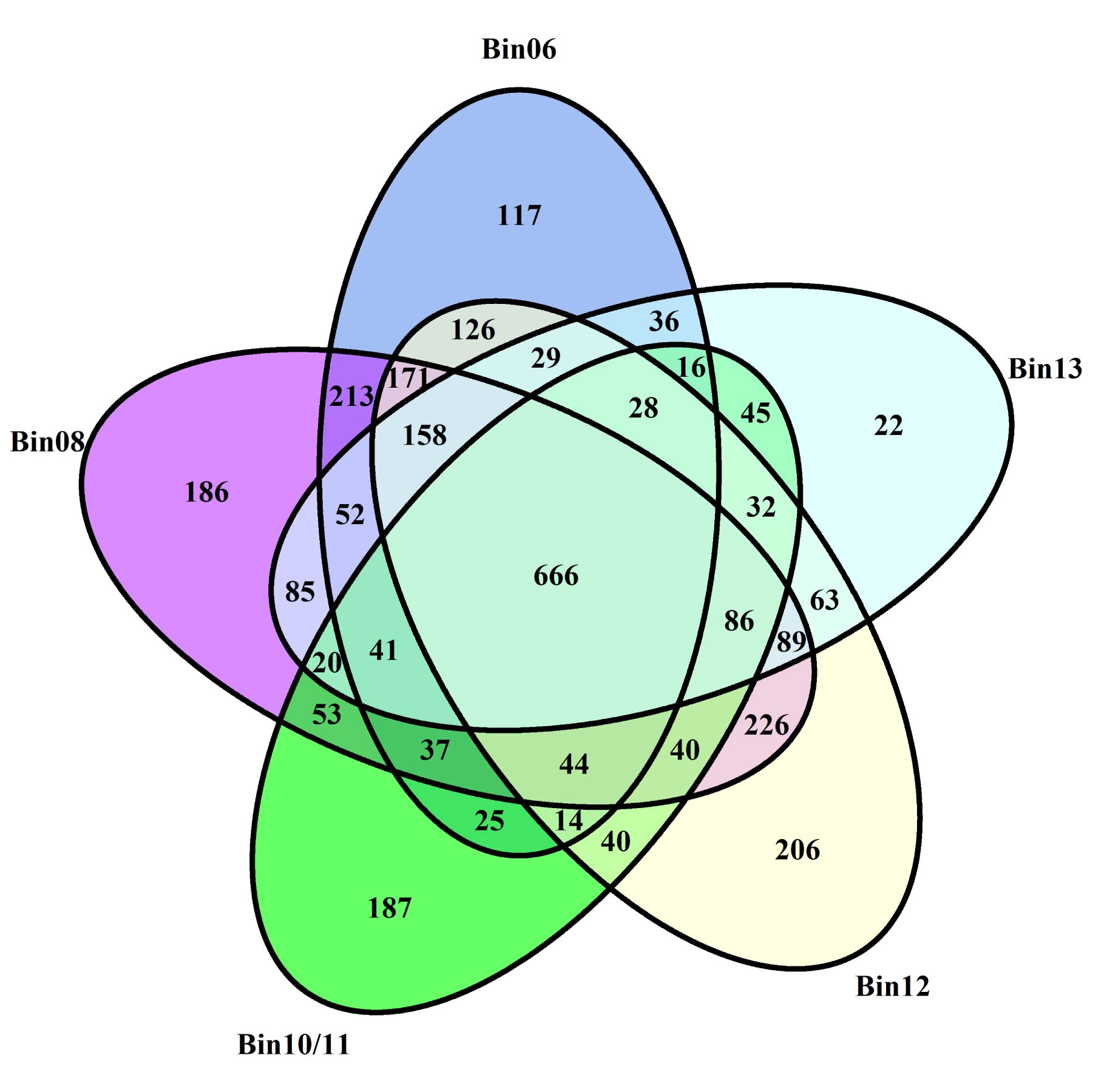


**Supplementary Figure S2.** Functions of the “Retain”, “Loss” and “Uniq” OGs of MAG Bin09. The three OG groups are referred to datasets in Figure 5. A: RNA processing and modification; C: Energy production and conversion; D: Cell cycle control, cell division, chromosome partitioning; E: Amino acid transport and metabolism; F: Nucleotide transport and metabolism; G: Carbohydrate transport and metabolism; H: Coenzyme transport and metabolism; J: Translation, ribosomal structure and biogenesis; K: Transcription; L: Replication, recombination and repair; M: Cell wall/membrane/envelope biogenesis; N: Cell motility; O: Posttranslational modification, protein turnover, chaperones; P: Inorganic ion transport and metabolism; Q: Secondary metabolites biosynthesis, transport and catabolism; S: Function unknown; T: Signal transduction mechanisms; U: Intracellular trafficking, secretion, and vesicular transport.


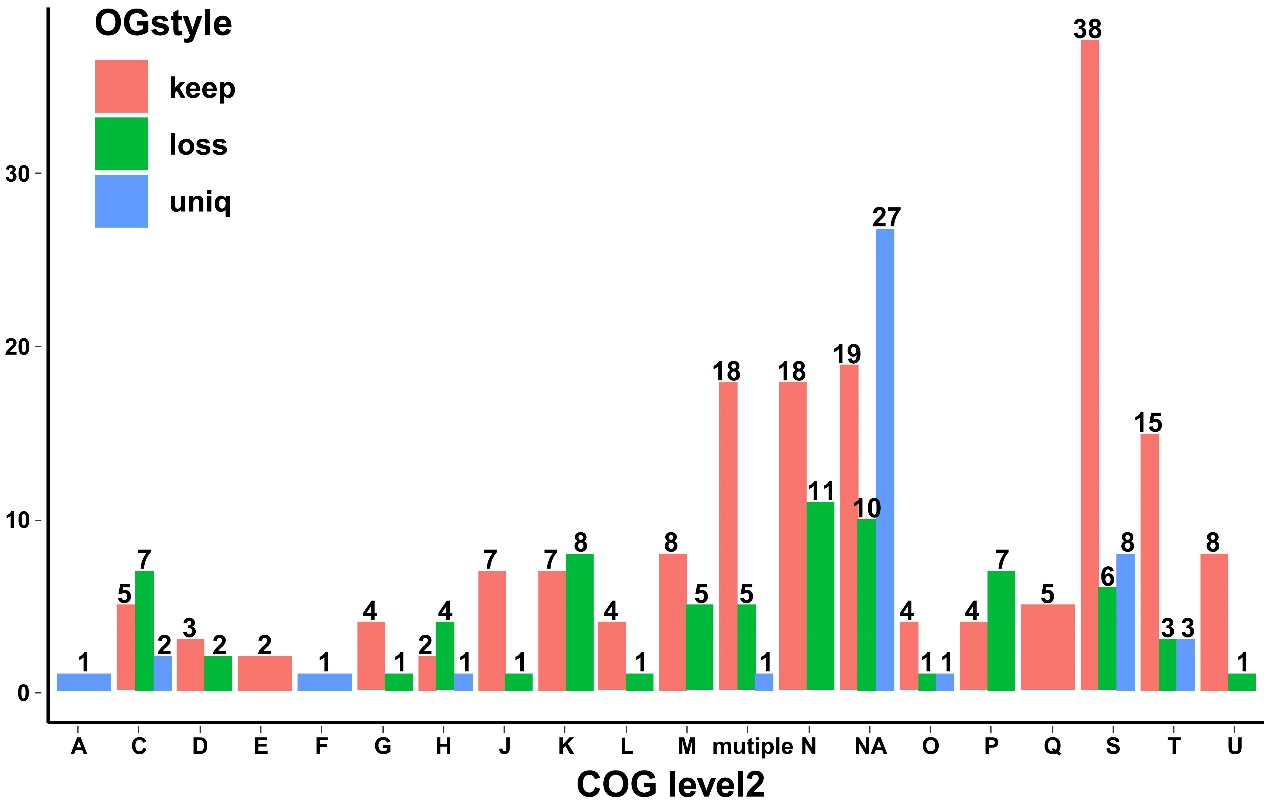


**Supplementary Figure S3.** The number of putative AMGs detected in predicted viruses. PF02562: phosphate starvation-inducible protein, *phoH*; PF13640: 2OG-Fe(II) oxygenase superfamily; PF01467: Cytidylyltransferase; EC:1.17.4.1: ribonucleotide reductase (RNR), *nrdA*/*nrdB*; PF05222: Alanine dehydrogenase/PNT, N-terminal domain; PF01451: Low molecular weight phosphotyrosine protein phosphatase; PF01503: Phosphoribosyl-ATP pyrophosphohydrolase; PF13469: Sulfotransferase family; K00254 : dihydroorotate dehydrogenase (EC:1.3.5.2); K00560: thymidylate synthase (EC:2.1.1.45); K09882: cobaltochelatase CobS (EC:6.6.1.2), *cobS*; M17: Catlytic type: Metallo; any N-terminal amino acid can be released from dipeptides and polypeptides, although there is a preference for leucine; bonds with proline in P1 are not cleaved; maximally active between pH 9 and 9.5; PF00266: Aminotransferase class-V; PF00406: Adenylate kinase; PF00719: Inorganic pyrophosphatase; PF01596: O-methyltransferase; PF02867: Ribonucleotide reductase, barrel domain; PF03328: HpcH/HpaI aldolase/citrate lyase family; PF04358: DsrC like protein; PF13759: Putative 2OG-Fe(II) oxygenase.


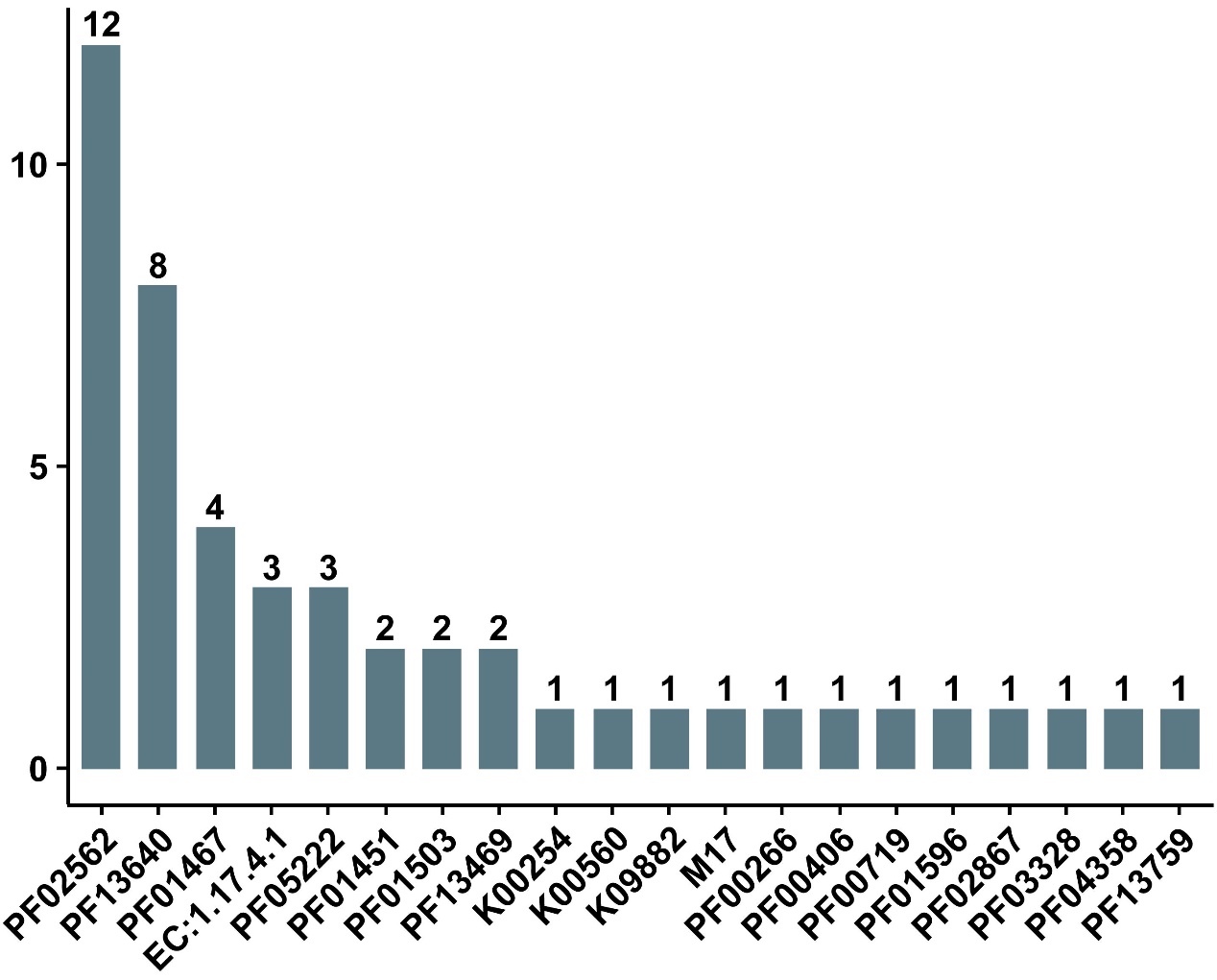

Supplement: Supplementary file 1 [file Supplementary_file_1.docx]
